# Developmental monitoring using caregiver reports in a resource-limited setting: the case of Kilifi, Kenya

**DOI:** 10.1111/j.1651-2227.2009.01561.x

**Published:** 2010-02

**Authors:** A Abubakar, P Holding, F Van de Vijver, G Bomu, A Van Baar

**Affiliations:** 1 Centre for Geographic Medicine Research – Coast, KEMRI/ Wellcome Trust Research ProgrammeKilifi, Kenya, and Tilburg UniversityTilburg, the Netherlands; 2 Centre for Geographic Medicine Research – Coast, KEMRI/Wellcome Trust Research ProgrammeKilifi, Kenya, and Africa Mental Health Foundation, and Case Western Reserve University, Kenya/Case Western Reserve UniversityCleveland, OH, USA; 3 Tilburg UniversityTilburg, Tilburg, the Netherlands, and North-West UniversityPotchefstroom, South Africa; 4 Centre for Geographic Medicine Research – Coast, KEMRI/ Wellcome Trust Research ProgrammeKilifi, Kenya; 5 Utrecht UniversityUtrecht, the Netherlands

**Keywords:** Africa, Caregiver reports, Children, Developmental monitoring

## Abstract

**Aim:**

The main aim of the current study was to evaluate the reliability, validity and acceptability of developmental monitoring using caregiver reports among mothers in a rural African setting.

**Methods:**

A structured interview for parents of children aged 24 months and less was developed through both participant consultation and a review of literature. The reliability and validity of the schedule was evaluated through a 10-month monitoring programme of 95 children, aged 2–10 months. The acceptability of the process was evaluated by studying retention rates and by organizing focus group discussions with participating mothers.

**Results:**

The structured interview ‘Developmental Milestones Checklist’ consisted of 66 items covering three broad domains of child functioning: motor, language and personal–social development. The interview yielded scores of developmental achievements that showed high internal consistency and excellent test–retest reliability. The results were sensitive to maturational changes and nutritional deficiencies. In addition, acceptable retention rates of approximately 80% were found. Participating mothers reported that they found the procedures both acceptable and beneficial.

**Conclusion:**

Developmental monitoring using caregiver report is a viable method to identify and monitor at-risk children in Sub-Saharan Africa.

## Introduction

Exposure to biological and environmental risk factors such as chronic poverty and its co-occurring factors can compromise child development, leading to a loss in cognitive and developmental potential (1). Early intervention can potentially ameliorate these negative effects leading to increased economic productivity, decreased delinquency and better school achievements later in life (2,3). Research indicates that early intervention programmes that focus on at-risk children in the first 2 years of life can be effective in improving IQ scores, with effect sizes ranging from 0.50 to 0.75 SD (4), but for an effective intervention, availability of adequate identification and monitoring procedures is necessary (5–7). Adequate assessment includes access to trained personnel and standardized assessment measures. Lack of access to both these elements significantly restricts the ability to implement early intervention monitoring programmes in developing countries (8,9). Resource constraints within the context of SSA suggest the need for approaches to identify at-risk children that do not require a high level of training, are cost effective and acceptable within the communities involved.

Assessment through the use of caregiver report may provide an effective approach to child monitoring, potentially bridging the gap in the lack of resources. Carefully constructed caregiver report instruments have demonstrated sound psychometric characteristics (10,11). Furthermore, caregiver reports cost approximately 10% of the administration costs of direct assessment or observation, while not requiring the high level of expertise in administration required by these latter methods (12).

The potential value of caregiver report instruments in low resource settings includes the relative ease of administration as well as the role that the data collection process can play in building a partnership between health care providers and caregivers (8). Inadequate knowledge of early child development among both caregivers and professionals may, however, compromise the validity of the reports elicited (13,14). The evidence provided by studies in low income countries that have used caregiver report for identifying at-risk children suggests that parents can provide reliable and valid information on their children’s development (13–15). However, none of the earlier studies in Africa has systematically evaluated how acceptable it would be for local communities to take part in an intense long-term follow-up of children to monitor their growth and development. The main aim of the current study was to evaluate the acceptability of developmental monitoring in a rural African setting using parental reports. Acceptability here refers to a combination of face validity of the tool, ease of administration and willingness of the mothers to engage in similar activities on a regular basis.

Specifically, we aimed at

- Developing a structured, comprehensive and easy to administer caregiver questionnaire on children’s developmental achievements;
- Evaluating the reliability and validity of responses to the caregiver questionnaire;
- Evaluating the acceptability of developmental monitoring among mothers.

## Methods

### Study site

The project was carried out in Kilifi, Kenya. Kilifi District has the second lowest per capita income in Kenya (16) with the majority of families dependent upon subsistence farming. Poverty in the district is characterized by low literacy levels, high infant mortality, high rates of malnutrition among under-fives, and endemic malaria (16). The study took place within a demarcated area in Kilifi District that undergoes active, four-monthly demographic surveillance, in which the births, deaths, and movement of individuals are recorded. The surveillance is carried out by the Centre for Geographic Medicine Research-Coast.

### Sampling

For the development of the questionnaire, the database of resident families was used to identify those with children in the target age range (3–24 months). Families were approached as required, for interview on trial versions of questions. A total of 63 families were involved in this iterative process of instrument development. The evaluation of the final questionnaire was carried out using families attending one of five government-run Mother Child Health (MCH) clinics spread across the study area. Four were satellite clinics, two in the northern and two in the southern study area, through which 70% of the sample were recruited, evenly distributed between clinics. The remaining 30% were recruited from the MCH clinic at the tertiary level government hospital, Kilifi District Hospital. Acquisition of participants was carried out over a period of 1 month, with sampling stratified to achieve equivalent numbers of boys and girls. Children were qualified for inclusion in the main study if they met the following criteria: (i) aged 2 to 10 months; (ii) parents spoke Kiswahili or one of the Mijikenda dialects as their primary language; (iii) families lived within the designated study areas; d) parent gave informed consent.

**Figure 1:**
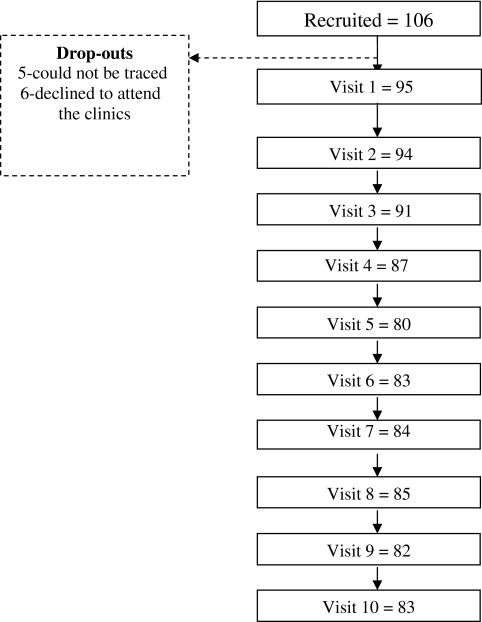
Recruitment and retention in the study.

An initial sample of 106 families was recruited for this first phase of data collection. Of these, 11 families did not enter the study (five had been recruited at the hospital, but gave incorrect or incomplete information to trace their households, while six changed their minds and withdrew the consent). Consequently, 95 families were involved in the first visit for developmental monitoring and were scheduled to attend the developmental monitoring process for 10 months. Figure 1 presents a summary of sample size at recruitment, patterns of attendance and attrition. At the first assessment point the children had a mean age of 7.13 months (SD = 2.54; range: 2.53–12.06) and they were followed up for 10 months until they had attained a mean age of 16.08 months (SD = 2.57; range 11.60–20.47). All mothers who attended the 10th and last visit of the monitoring programme (n=83) were invited to attend a focus group discussion in order to evaluate the programme. Approximately 93% (n=72) of the invited mothers attended.

### Measures

The Developmental Milestones Checklist (DMC) was administered alongside four other measures. Additional measures administered included: *i) Height*– measured lying down, using a Rollameter. The visitor assisted by the mother took the measures following the CDC recommended protocol for taking height. *ii) Weight*– taken, undressed, on a SECA Digital Scale. The children were weighed three times and records of weight were taken until consistent results were obtained across two of the measures to at least one decimal point. *iii) Maternal education*. Mothers were asked to indicate the number of years they had attended formal education. A dichotomous variable, schooled vs unschooled, was created. Schooling was defined as having completed at least 1 year of formal education. *iv) Kilifi Developmental Inventory (KDI),* a locally developed measure of psychomotor functioning was administered through interacting with the child (21). The KDI measures two positively correlated factors, Locomotor Skills and Eye-Hand Coordination. Scores on the two factors can be added to provide a single overall score, labelled Psychomotor Skills.

### Procedures

#### The development of the Developmental Milestones Checklist (DMC)

The aim was to develop a questionnaire based on caregiver report through a three-stage approach which involved (i) defining the construct(s) to be measured, (ii) creating an item pool and (iii) selecting a final list of items;

- *Construct definition*: we aimed at developing a measure that assesses developmental outcomes in the first 2 years of life. Two steps were used to define the constructs assessed. First, a review of the literature on existing measures of early development was carried out, specifically studying the Griffiths Mental Developmental Scale for Infants (GMDS) (17) and the Vineland Adaptive Behaviour Scale (18). Second, the target community was consulted to evaluate face validity of the identified items and to identify new items on potentially important achievements. Consultation with the target community was carried out in a series of 6 focus groups (4 groups were made up of mothers randomly selected from the community, 1 consisted of teachers and 1 of paediatric nurses). In these focus groups participants were asked to identify developmental changes that children experience in the first 3 years of life. For every mentioned skill, the moderator inquired about the age at which children were expected to acquire the skill. A qualitative check was carried out to confirm that items mentioned by parents closely mirror what we had identified through our literature search, thus providing evidence for face validity.
- *Item pool creation:* An initial item pool of 104 questions was drawn, largely based on the GMDS and Vineland Adaptive Behaviour Scale. Items assessed locomotor, fine motor, language and personal–social development. All identified items were translated and back translated by two people who were fluent in both English and Swahili. The approach to scoring and interviewing was based on a questionnaire that had been developed elsewhere in East Africa (19) and subsequently used successfully in Kilifi with older children (20).
- *Piloting, item selection and training of the community health worker:* Responses were evaluated by a panel consisting of six early childhood assessors and two psychologists using the following criteria: (i) clarity (any item that elicited ambiguous responses was removed); (ii) cultural appropriateness (items reflecting an activity or behaviour familiar to the respondents); (iii) age appropriateness; (iv) ease of expression in the local language. Based on this process, 38 items were excluded. The community health worker had secondary level education and extensive experience in interviewing techniques, but had no prior training in child development. The training in developmental monitoring for the community health worker took 2 days.

To evaluate the reliability and validity of the developed measure, mothers were invited to bring their children to a clinic nearest to their homes at the appointed date. During this visit, a trained community health worker completed the items of the DMC with the caregiver in a face-to-face oral interview. In addition, the anthropometric measures were taken. When a mother failed to come for the scheduled clinic visit, she was visited at home. Based on the age at recruitment, children also underwent an appointed home-based assessment when they were 6, 9, or 12 months old. During the home visit, a trained developmental assessor administered the KDI, that incorporates observation and direct assessment (21). Data of the psychomotor skills were used to compare the relationship between caregiver reports and the observed skills of the children.

#### Programme evaluation

The main aim of this phase was to evaluate the developmental monitoring programme. Five focus groups were held, one at each clinic. Each focus group was attended by a moderator, a note taker and an assistant drawn from the study team. Sessions were audio taped and hand-written notes were taken. The main research questions posed to the focus groups were: (i) what were the perceived benefits and liabilities of study participation? (ii) What were the factors that either facilitated or hindered participation in the study?

### Data management and analysis

Data were double entered in FoxPro and verified before being transferred to SPSS (version 12) (SPSS Inc., Chicago, IL, USA) for analysis. Cronbach’s alpha and Intraclass Correlation Coefficients (ICC) were used to evaluate reliability. Validity and sensitivity were evaluated using Pearson Product Moment Correlations and Analysis of Variance (ANOVA). Weight-for-age (WAZ) and height-for-age (HAZ) standards were generated using the WHO software (World Health Organization, Geneva, Switzerland) for assessing growth and development (22).

Using notes taken during the focus group discussions (FGD), transcripts from FGD were prepared by the facilitators, translated into Kiswahili where there was a need and entered into Microsoft word documents. Based on an a priori decision, a thematic framework focusing on perceived benefits, perceived liabilities and factors contributing to attendance and non-attendance was developed, to analyse the information generated from the focus group discussions. The themes were chosen with the assumption that they allowed us to evaluate the acceptability of the programme in the community. Two people (AA and an independent person) carried out the thematic analysis separately, the results were discussed and consensus reached where there were disagreements.

## Results

The final questionnaire, the *Developmental Milestones Checklist (*DMC), monitors development in infants aged 3–24 months in three main domains, motor, language and social–emotional development. The 66 constituent items are administered as a structured interview (see Table S1 for a sample of the questions). The items are scored on a three-point Likert scale (0: not observed, 1: emergent – defined as child has been observed to attempt to perform the skill in the last month, and 2: established behaviour – defined as the child has been observed to perform the skill for more than a month). The questionnaire takes approximately 15 min to administer.

### Reliability

Estimates of *internal consistency* for the DMC, based on coefficient alpha (n=95), were all excellent (Motor: α = 0.91; Personal–social: α = 0.87; Full Scale: α = 0.94) except for Language;α = 0.62, which is a fair value according to standards described by Ciccheti (23). *Retest reliability* was estimated by computing Intraclass Correlation Coefficients (consistency); the values were similar (Motor: 0.88; Personal–social: 0.67; Language: 0.66; Full Scale: 0.85).

The *dimensionality* of the subscale scores was studied in a principal component analysis. The scores from the three subscales yielded a strong first component, which accounted for 75% of the variance (Eigenvalue = 2.25). Factor loadings were as follows: motor 0.92, language 0.79, and Personal–social 0.89. These results support the use of a summated score as an overall index.

### Gender and performance

A *t*-test indicated that there were no significant *gender differences* in the developmental scores *t*(93) = −0.76, p=0.45. The absence of gender differences was confirmed at the sub-scale level (Motor: *t*(93) = −0.48, p=0.63; Language: *t*(93) = −0.82, p=0.42; Personal–social: *t*(93) = −0.74, p=0.46).

### Sensitivity to age

The age sensitivity of the DMC scores was investigated by correlation these with age. Strong correlations were found between age and the developmental score (r(95) = 0.82, p<0.001), explaining approximately 67% of the variance; significant relationships were found for all subscales (Motor: r(95) = 0.88, p<0.001; Personal–social: r(95) =0.65, p<0.001; Language: r(95) = 0.57, p<0.001).

**Figure 2:**
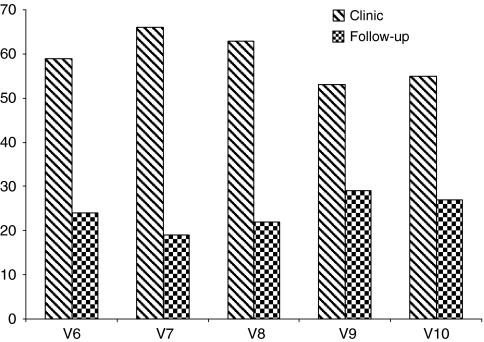
Standardized means of the total developmental score and their 95% confidence intervals according to caregiver report across all the ten time points. NB: The X–axis is presented with the mean age and standard deviation for each time point.

Scores of the children who were seen at all 10-time points (n=69) were used to evaluate the sensitivity of the DMC to *maturational changes*. Changes in scores across time were tested in a repeated measures analysis of variance, with time points as independent variables and scale scores as dependent variables (see Fig. 2 for a depiction of the changes in means over time). The value of Wilks’ Lambda indicated significant change in scores over the ten-month period in all scales; all univariate follow-up tests showed a significant increase in achievements of the children (Developmental score: *F*(9, 60) = 199.63, p<0.01; Motor: *F*(9, 60) = 140.88, p<0.01; Language: *F*(9, 60) = 99.31, p<0.01; Personal–social: *F*(9, 60) = 64.06, p<0.01.)

### Concurrent Validity

Caregiver reports were correlated with the performance-based assessment of scores of psychomotor functioning using the KDI (21) to establish the validity of caregiver reports. A significant correlation was observed between the total score of the caregiver reports and the KDI scores, r(87) = 0.80, p*<*0.001. The strongest relationship was observed between the motor subscales in caregiver report and the locomotor subscale in the KDI, r(87) = 0.84, p*<*0.001, see Table 1.

**Table 1:** Correlations between the psychomotor scales of KDI and caregiver report

	Psychomotor scores		
Caregiver report	Locomotor	Eye-hand	Psychomotor
Motor	0.84**	0.73**	0.83**
Language	0.63**	0.60**	0.64**
Personal–social	0.55**	0.50**	0.55**
Total score	0.80**	0.72**	0.80**

** p<0.01.

### Sensitivity to group differences

Sensitivity of the measure was evaluated for *stunting (*defined as having a score below −2 SD of the WHO HAZ standards*)* vs children with normal HAZ. Children in the stunted group showed a significantly poorer performance than the other children, *F*(1, 93) = 17.58, p<0.001, η^2^ = 0.16. Similar results were observed for each scale (Motor: *F*(1, 93) = 8.99, p<0.001, η^2^ = 0.09; Language: *F*(1, 93) = 7.77, p<0.05, η^2^ = 0.07; Personal–social: *F*(1, 93) = 10.37, p<0.001, η^2^ = 0.10). The results of children underweight (defined as having a score below −2 SD of the WHO WAZ standards) were compared with those of normal weight children. The former group showed a significantly poorer performance *F*(1, 93) = 19.30, p<0.001, η^2^ = 0.17. Similar results were observed for each of the scales (Motor: *F*(1, 93) = 13.67, p<0.001, η^2^ = 0.13; Language: *F*(1, 93) = 6.21, p<0.05, η^2^ = 0.06; Personal–social: *F*(1, 93) = 11.23, p<0.001, η^2^ = 0.11). In addition, children of mothers who were not schooled did not differ in their developmental performance score on the questionnaire from children of schooled mothers (developmental score: *F*(1, 93) = 2.10, p<0.15; Motor: *F*(1, 93) = 0.28, p<0.60; Language: *F*(1, 93) = 0.48, p<0.50; Personal–social: *F*(1, 93) = 3.36, p<0.07).

### Retention rates

**Figure 3:**
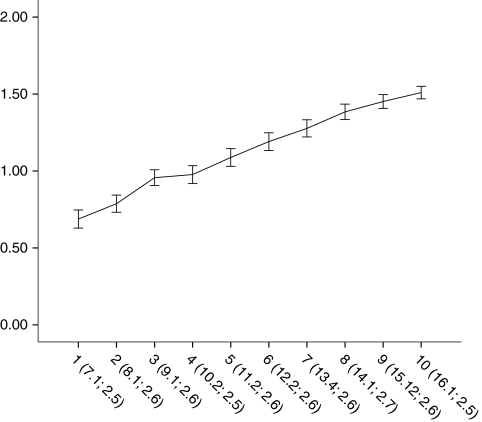
The number of parents attending clinic visits and parents who had to be followed up at home from the sixth to the tenth visit.

Of the initial 106 recruited, data from 83 mothers were available at the end of the ten months period. Figure 1 presents pattern of attendance. Numbers fluctuate due to a pattern of non-attendance in certain months and a return to the study the next months. Most of those dropping out for a visit and coming back later did this due to travel outside of the study area. The final attendance of 83 out of the original 106 represents an attrition rate of 21%*.* An analysis of the attrition patterns indicated no significant differences in age (*t*(95) = −0.83, p=0.42), gender (χ^2^(1, n=93) = 0.13, p=0.72), and initial developmental status (*t*(95) = −0.55, p=0.58) of children who dropped out after attending the first session compared to those who completed the study. In addition, each mother was given a date to come for monitoring at a designated clinic. However, a significant percentage did not come for their scheduled visits at the clinic and we had to collect the data at their home. Figure 3 presents the pattern of clinic and home visits for the last five waves.

### Perceived benefits

The mothers reported several perceived benefits including increased awareness of their child’s developmental stages, the need to stimulate and encourage their child’s growth and the toys and play activities they could use to provide further stimulation. Table S2 presents a summary of the responses.

### Factors encouraging agreement to participate in longer term monitoring

Given the time commitment required, we asked what had prompted them to consent to participate initially and to continue to attend. For some, both consent and attendance was prompted by curiosity about what would happen in the course of the study, whereas other mothers had a desire not to appear rude by declining to take part. A third group did so at the insistence of their husbands. Continued attendance was also encouraged by the belief that the developmental monitoring was more useful than the simple growth monitoring carried out at standard clinic visits; online Table S3 presents a detailed summary of the reasons given for participation as well as the costs and benefits.

### Perceived liabilities

No group reported experiencing the programme as risky or harmful for the participating families, although there was some concern about the burden of time and the interruption of their daily schedule (see online Table S2).

### Factors hindering participation

The most commonly mentioned factors were incompatibility with other obligations and having to travel outside the study area for brief visits with relatives (see online Table S3).

## Discussion

### Development, reliability and validity of caregiver reports

Using a mixed-method approach, we developed a parent report questionnaire, that showed good psychometric properties; with high internal consistency and test–retest reliability. Furthermore, both face and concurrent validity were supported. The sensitivity of the instrument was also demonstrated by the significant association between anthropometric status and all aspects of reported development. The sound psychometric properties and the ease of administration by people with limited previous training in child development attest to the suitability of the instrument for use in similar settings.

Our study did not allow for a full evaluation of the clinical utility of the DMC and we are as yet unable to furnish guidelines on how the procedure can be used to identify children at risk. Future efforts will aim at developing cut-off scores through investigations with clinical samples.

### Acceptability of an intensive follow-up programme

The acceptability of the programme was inferred from the retention rates and further supported by satisfaction with the benefits of participation, as expressed by the mothers. In particular, mothers expressed a positive attitude towards the opportunity to learn about development and monitor their own children. This finding is an important indicator of a potential programme’s success and replicates the reaction to nutritional monitoring in other settings in SSA (24,25).

However, the use of the same community health visitor to run the programme and to act as moderator of the evaluation FGD’s may have influenced the mothers to temper their criticisms. Her presence as moderator and the self-selecting nature of the group, in that they were the mothers who had chosen to remain in the programme, may have led to a more positive evaluation, despite our encouragements of the mothers to provide critical comments.

The groups did indeed report negative feelings. One concerned the time burden of a programme that requires monthly visits. This observation suggests that for children for whom no concern over their development exists, a longer time interval between visits might be more acceptable. Another option would be to develop a home-based developmental monitoring programme, which would avoid families travelling to the clinic. This option would require a higher staffing level of community health workers in a programme and may therefore significantly increase the cost of its delivery. We only visited the ‘no shows’ at home, but not if the mothers indicated that they no longer wished to take part in the study. Selective home-based follow up may be a more affordable alternative to an entirely home based programme and enable consistent monitoring of at-risk cases.

Some participants freely shared that their continued participation was not due to personal preference, but occurred because of either family pressure (i.e. the father’s enthusiasm) or a desire to be polite to the community health worker, that is not to appear rude by dropping out. Future efforts need to investigate how communication between programme developers, the participant and the family of the participant can be enhanced to avoid a situation where participation is maintained due to real or perceived pressure.

Taken together, these results indicate that caregiver reports of children’s achievement levels as elicited by the Developmental Milestones Checklist provides a reliable and valid methodology to gather information for identifying potentially at-risk children. Based on the findings of this first study, it can be concluded that it is possible to design a developmental monitoring programme for a resource-limited setting that is appropriate for the resources available and acceptable to the mothers.

## SUPPORTING INFORMATION

**Table S1** Description of the Items in the Developmental Milestones Checklist (Readers interested in accessing the full questionnaire can contact the first author)

**Table S2** Perceived Benefits and Liabilities in Taking Part in the Infant Monitoring Programme

**Table S3** Reasons for Participating in the Programme and for Missing Appointments
