# Supplementary material for: Developmental monitoring using caregiver reports in a resource-limited setting: the case of Kilifi, Kenya
Source: Acta Paediatr. 2010 Feb;99(2):291–7. doi: 10.1111/j.1651-2227.2009.01561.x (PMC2814084; doi:10.1111/j.1651-2227.2009.01561.x)
Supplement: Supplementary file 1 [file apa0099-0291-SD1.doc]

*Online supplementary Table 1: Description of the Items in the Developmental Milestones Checklist[[1]](#footnote-2)*

| Name of subscale | Skills assessed | Sample items |
| --- | --- | --- |
| Motor (N = 28) | Head control, sitting, crawling, walking, running, kicking, throwing, reaching, object manipulation, picking and writing. | Sits supported |
|  |  | Stands holding on to furniture or other objects |
|  |  | Climbs on to a low chair |
|  |  | Reaches and grasp objects |
|  |  | Scribbles with a pen |
| Language (N = 11) | Pre-speech, gesture use, use of single words, object naming and recognition, | Repeats syllables in strings |
|  |  | Repeats vowels in strings |
|  |  | Uses gestures |
|  |  | Can identify familiar objects |
|  |  | Names familiar objects |
| Personal-social (N = 27) | Reaction to others, recognition of others, self-recognition, daily living skills. | Maintains eye contact |
|  |  | Reacts to own name |
|  |  | Tries to help dress arms into a shirt |
|  |  | Joins other children in play |
|  |  | Bowel control complete, rarely dirty by day |

Online supplementary material Table 2

Perceived Benefits and Liabilities in Taking Part in the Infant Monitoring Programme

| Theme | Sub-themes | Examples of supporting statement | *N* |
| --- | --- | --- | --- |
| Perceived Benefits | | |  |
|  | Children experienced accelerated growth | ‘…I suspect the items you gave my child or the activities he participated in stimulated his mental growth…’ | 5 |
|  | Increased awareness of the need to stimulate child development | *‘‘*The important thing I learnt is that when you give birth you need to teach them different things, need to try and see if they will learn…’ | 4 |
|  | Actively monitored child’s development | ‘As the months passed you know that a child must be monitored... I mean you must understand them’ | 4 |
|  | Increased awareness how to stimulate child development. | ‘ ..that when the child speaks to you, then you need to respond… even if you do not understand them try to respond ’ | 4 |
|  | Increased awareness of child’s developmental stages. | ‘Now we know more… child can remember…. We had not monitored this despite having given birth to other children… this is not my first born, but did not know (a lot). So in my life I received an eye opener’. | 4 |
|  | Encouraged to participate in play activities | ‘(Sometimes even when not visited (team has not gone to assess the child) we try out the activities so that we can also grow mentally (improve ourselves)…..’ | 2 |
|  | Increased awareness of their teaching role | ‘.learnt… we are like teachers....’ | 1 |
|  | Free medical treatment when the child was ill | ‘Since I had this baby, he has always had (health) problems, then you came to him, you said I bring to your clinic you try to help... I came and child is better now’ | 1 |
|  | Child got new and exciting experiences | ‘Child got a chance and used to travelling by car to Kilifi (clinic) | 1 |
|  | Learnt of child’s nutritional needs | ‘Only learnt about food (feeding the child)’. | 1 |
|  | Detailed and accurate measurement of growth | ‘So important for where we go (Antenatal Clinic) they do not measure head, arm or length’ | 1 |
| Perceived Liabilities | | |  |
|  | None | ‘Nothing bad observed’ | 5 |
|  | Did not approve of some of assessment procedures | ‘… this idea of coming we talk and then you look around the house (observe the home environment and physical set up’. | 3 |
|  | Time consuming | ’May be the fact that we had to sit for many hours; sometimes one had to wait half an hour’ | 2 |
|  | Husband annoyed by her participation | ‘When you come (to the home) I got scared because I had told him (husband) I was no longer attending the clinics) | 2 |
|  | Interruption of schedule | ‘Problem is that one has to break (disrupt) daily schedule to come to clinic’ | 2 |
|  | Unannounced visits i.e. visits without prior appointment | ‘I felt disturbed because the first time (you visited) I was not aware … (I had) gone out with daily business (I) come back and was told you were there… I was unhappy …’ | 1 |
|  | Fatigue in responding to the same set of questions | ‘The questions were too many’ | 1 |

*N*: Number of focus groups in which the issue was raised (total *N* = 5)

Online supplementary material Table 3

Reasons for Participating in the Programme and for Missing Appointments

| Themes | Sub-themes | Examples of statement | *N* |
| --- | --- | --- | --- |
| Reasons for taking part | | | |
|  | Curiosity | ‘Just wanted to know what will happen…’ | 3 |
|  | Encouraged and sometimes compelled by spouse | ‘If it was nor for my husband I would have stopped long time... but he was so enthusiastic, that I wondered why some months ago you did not invite him’ | 2 |
|  | Voluntary participation | ‘…because from the very start the study was properly explained and we made the decision to take part so we had to continue’ | 1 |
|  | Attends to avoid being perceived as rude | ‘if I did not attend you would have thought I am rude’ | 1 |
| Reasons for missing appointments | | |  |
|  | Attending to another obligation | ‘I was nursing a sick relative’ | 4 |
|  | Forgot the dates | ‘had no one around to read for me the dates for the next appointment’ | 2 |
|  | No one to assist care for the other children | ‘I did not attend because I had no one to assist me’ | 2 |
|  | Mother or child was ill | ‘I missed because I was ill… even when you followed me to the house I was still having a fever’ | 2 |
|  | Travelling | ‘I had to travel elsewhere at that time’ | 1 |
|  | Busy | ‘I had a lot of other things to attend to’ | 1 |
|  | Spousal refusal | ‘I did not attend because my husband told me not to attend’ | 1 |
|  | Notice was short | ‘I was given the fare/appointment? so abruptly that is why you did not see me’ | 1 |

*N*: Number of focus groups in which the issue was raised (total *N* = 5).

1. Readers interested in accessing the full questionnaire can contact the first author. [↑](#footnote-ref-2)
